# How a National Organization Works in Partnership With People Who Have Lived Experience in Mental Health Improvement Programs: Protocol for an Exploratory Case Study

**DOI:** 10.2196/51779

**Published:** 2024-04-19

**Authors:** Ciara Robertson, Carina Hibberd, Ashley Shepherd, Gordon Johnston

**Affiliations:** 1 Faculty of Health Sciences and Sport University of Stirling Stirling United Kingdom

**Keywords:** partnership, engagement, case study, mental health, improvement, national program, quality improvement

## Abstract

**Background:**

This is a research proposal for a case study to explore how a national organization works in partnership with people with lived experience in national mental health improvement programs. Quality improvement is considered a key solution to addressing challenges within health care, and in Scotland, there are significant efforts to use quality improvement as a means of improving health and social care delivery. In 2016, Healthcare Improvement Scotland (HIS) established the improvement hub, whose purpose is to lead national improvement programs that use a range of approaches to support teams and services. Working in partnership with people with lived experience is recognized as a key component of such improvement work. There is, however, little understanding of how this is manifested in practice in national organizations. To address gaps in evidence and strengthen a consistent approach, a greater understanding is required to improve partnership working.

**Objective:**

The aim of this study is to better understand how a national organization works in partnership with people who have lived experience with improvement programs in mental health services, exploring people’s experiences of partnership working in a national organization. An exploratory case study approach will be used to address the research questions in relation to the Personality Disorder (PD) Improvement Programme: (1) How is partnership working described in the PD Improvement Programme? (2) How is partnership working manifested in practice in the PD Improvement Programme? and (3) What factors influence partnership working in the PD Improvement Programme?

**Methods:**

An exploratory case study approach will be used in relation to the PD Improvement Programme, led by HIS. This research will explore how partnership working with people with lived experience is described and manifested in practice, outlining factors influencing partnership working. Data will be gathered from various qualitative sources, and analysis will deepen an understanding of partnership working.

**Results:**

This study is part of a clinical doctorate program at the University of Stirling and is unfunded. Data collection was completed in October 2023; analysis is expected to be completed and results will be published in January 2025.

**Conclusions:**

This study will produce new knowledge on ways of working with people with lived experience and will have practical implications for all improvement-focused interventions. Although the main focus of the study is on national improvement programs, it is anticipated that this study will contribute to the understanding of how all national public service organizations work in partnership with people with lived experience of mental health care.

**International Registered Report Identifier (IRRID):**

DERR1-10.2196/51779

## Introduction

### Overview

The need to improve quality in mental health (MH) care is widely recognized, in response to both long-standing problems and more contemporary pressures [1,2]. For several years, quality improvement (QI) has been considered a key solution to many health care challenges, supporting the design and delivery of services. Over the last decade, there has been a significant effort to use QI within health care settings, including the introduction of national organizations to lead improvement programs.

There are several national organizations in Scotland with an improvement focus, including the Centre for Sustainable Delivery, the Health and Social Care Alliance Scotland, the Improvement Service, and Healthcare Improvement Scotland (HIS). In 2016, HIS established the improvement hub (ihub), whose purpose is to enable health and care systems to apply improvement methodologies to the design and implementation of changes that deliver sustainable improvements in the health and well-being outcomes of people in Scotland [3]. The ihub within HIS is uniquely placed with a focus on improvement support for those delivering health and social care across Scotland, including MH services.

**Figure 1:**
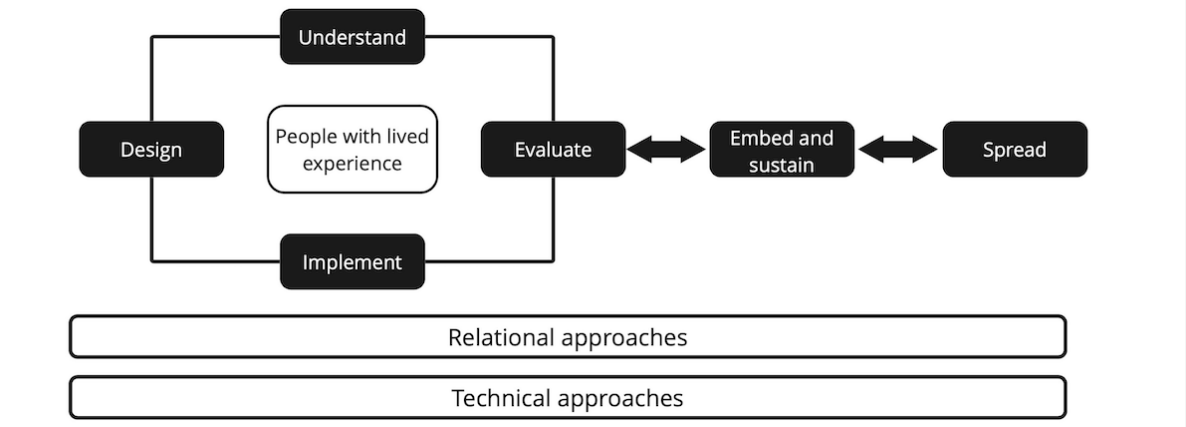
Components of improvement programmes, adapted from Healthcare Improvement Scotland's Framework for Planned Improvement.

Work within the ihub is delivered through improvement programs that use a range of theories and techniques to support teams and services through an improvement journey. National improvement programs have an important role to play in health care. However, there are challenges within centrally led programs that require sensitive understanding and management [4]. The development of improvement programs recognizes growing evidence that the impact of QI in health care is mixed and of poor quality [5], and there is a need to reconceptualize improvement efforts in response to the evidence base [6]. In order to address some concerns within the literature, the ihub has outlined a broad approach to improvement that forms the basis of their improvement programs. The core components of improvement programs within the ihub are described in the framework for planned improvement (Figure 1 [3]), which outlines the stages of improvement work. In the Framework for Planned Improvement, the initial focus is on understanding the system and designing, implementing, and evaluating changes, with people with lived experience at the center of this work. People with lived experience include people who have lived or living experience, their families, caregivers, and supporters. Improvement programs then aim to embed and sustain successful change within practice and spread the learning to other areas. Underpinning the framework is the recognition of the importance of the relational aspect of change and the use of technical QI approaches, including the model for improvement.

A key principle to improvement is working in partnership with others in the system, including other agencies, people with lived experience, and frontline staff. In Scotland, a seminal paper by Christie [7] recommended that there should be a stronger partnership working with people and communities in the design and delivery of services they use, including those involved in health care improvement. There is a growing evidence base supporting the need to work with people with lived experience in health care improvement. People with lived experience have a key role to play in understanding problems and identifying solutions to ensure change delivers outcomes that make a difference to patients [8]. Working with people with lived experience in improvement initiatives can strengthen and enrich the organizational agenda for improvement in health care [9] and should be seen as a core component of all improvement programs. Within MH services, people with lived experience should be able to participate in the development of policies to improve MH systems [10] and should therefore be involved in health care improvement initiatives. Working with people with lived experience should be based on authentic, interdependent partnership work [6], which will improve the quality and value of services.

Despite the recognition that working with people with lived experience is central to improvement-focused work, there are a number of challenges and a lack of critical examination of partnership working within the health care improvement literature [11]. There is a lack of understanding of the phenomenon of partnership working, including the mechanisms of partnership working, organizational features supporting partnership working (eg, leadership), and the impact and outcomes achieved from working with people with lived experience [11,12]. There is also little understanding in the literature of how working with people with lived experience is manifested in practice in national organizations [13].

This research will explore how a national organization works in partnership with people with lived experience in a MH improvement program. This research will focus on one improvement program—the Personality Disorder (PD) Improvement Programme within HIS’ ihub. The PD Improvement Programme is a commissioned piece of work funded by the Scottish Government to understand the current service provision in Scotland for people with a diagnosis of PD and identify the key opportunities for improvement. This research will use a case study approach to explore how partnership working is planned, conceptualized, and manifested in practice within the PD Improvement Programme.

### Objectives

The aim of this study is to better understand how a national organization works in partnership with people who have lived experience with improvement programs in MH services, exploring people’s experiences of partnership working in a national organization. An exploratory case study approach will be used to address the research questions in relation to the PD Improvement Programme:

1. How is partnership working described in the PD Improvement Programme?
2. How is partnership working manifested in practice in the PD Improvement Programme?
3. What factors influence partnership working in the PD Improvement Programme?

This research will consist of 2 phases. The first phase will address the first 2 research questions through document analysis and observations of meetings within the early stages of the PD Improvement Programme. Semistructured interviews will be carried out in the second phase of this research to explore participants’ experiences of partnership working, addressing the third research question.

### Benefits of This Research

It is anticipated that the findings of this research will contribute to an understanding of partnership working in national organizations and will be used to identify a framework for partnership working so that partnership working can be improved across the organization and other national organizations.

## Methods

### Overview

In order to address the research aim, it is appropriate to use case study methodology. A case study approach is appropriate when the focus of the study is on how and why questions; the behavior of participants will not be changed; the context is relevant to the phenomenon studied; and when there are unclear boundaries between phenomenon and context [14]. Partnership working sits within the wider context, and case study methodology is well placed to understand relationships between context and intervention [15], with partnership working conceptualized as the intervention in this research. A case study approach will enable a holistic exploration of the complex social processes and mechanisms underpinning partnership working within QI [16]. Data will be collected from a wide range of qualitative sources, including document data, participant observations, and semistructured interviews.

### Case Study Design

The DESCARTE model [17] will be used in this research to inform the design, conduct, and reporting of the case study. There are 3 stages to this model: the situation of the research and the researcher, determining the components of the case study design, and data analysis.

### Situation of the Research and the Researcher

In designing case study research, it has been recommended that the researcher state explicitly their informing philosophical approach, situation of “Self” within the research, and any ethical considerations to outline the position of research and the researcher [17].

The lead researcher is currently working as part of the improvement team within HIS and therefore will be considered an insider researcher. Although this position may support access to naturalistic data and respondents, there is a risk that there may be conflict between the researcher and participants who have professional relationships, and a risk that respondents may change their behavior or responses due to this relationship [18]. This will increase the risk of bias within the research, and strategies should be used throughout the different stages of the research process to reduce these risks [19]. For this study, strategies will include planning the interview process, using research diaries, reflection, and ongoing monitoring with the supervisory team. The lead researcher will also work closely with a public partner at key stages of this research. Public partners are volunteers who HIS trains and supports to provide a public perspective to their work, and a public partner with lived experience of mental illness will be involved at several stages of this research.

### Components of the Case Study

Although case study research can have a level of creativity and flexibility—where the researcher may choose epistemologies and theories suited to their preferences and the nature of the inquiry, clear descriptions of paradigms, theories, and methods should be provided to demonstrate rigor [20]. These will be described to outline the main components of the case study.

#### Binding the Case

First, it is important to identify what the case will be and set clear parameters or boundaries to ensure the study has a clear and reasonable scope—a process referred to as binding [21]. The parameters of this study will be determined by definition and context; for this research, the case will consist of the PD Improvement Programme within HIS. Early involvement of people with lived experience in the conceptual stages of improvement work has been highlighted to ensure meaningful involvement with influence and impact [22]. The PD Improvement Programme is the first commissioned work for HIS to improve the understanding of the context of service provision for people with a diagnosis of PD across Scotland. The program will include working with people with lived experience and frontline staff working in clinical roles. The commission is from the Scottish Government and will run between June 2021 and March 2023. This case study will follow the PD Improvement Programme during the current stage of the program: creating the conditions and understanding the system. This stage will involve establishing the program and working practices for working in partnership during the PD Improvement Programme. The parameter for this case is to explore working in partnership with people with lived experience and will not include exploration of wider partnerships working in this program.

#### Type of Case Study

Exploratory case studies can be used to explore situations in which the intervention being researched does not have a clear, single set of outcomes [21]. Given the diversity within QI and the complexity of partnership work, an exploratory approach is considered appropriate.

#### Design

In phase 1 of this case study, data will be collected from organizational documents, followed by nonparticipant observations of key program meetings. This data will help explore how partnership working is described, defined, and manifested in practice. This will be followed in phase 2 by semistructured interviews with key participants to explore their experiences of partnership working in the program.

#### Phase 1: Document Data

In the first phase of data collection, analysis of organizational documents will be used to provide an understanding of plans, infrastructure, and frameworks used to support partnerships working with people with lived experience. It is anticipated that documents may include commission agreements, planning papers, minutes of key meetings, presentations or diagrams describing the program infrastructure, and partnerships working in the program. Further documents relevant to the study may emerge and will be included as appropriate. Access to these documents will be through the program lead within HIS.

As there is no agreed definition of partnership working, documents will be analyzed for any description of partnership, which may include terms such as involvement, participation, engagement, and empowerment. The content of documents will be analyzed, including the document, author, date, description of partnership working, and any actions taken or recommendations. Meetings with the public partner will be agreed upon to discuss the data analysis and the identification of themes at each stage of the data analysis.

Themes developed from the document review will be included in the structure of observations and used to develop the interview proforma in the following phases of the research.

#### Phase 1: Nonparticipant Observations

Following document analysis, nonparticipant observations of PD Improvement Programme meetings will be used to gather data on how partnership working with people with lived experience in the program is manifested in practice. Meetings observed will be chosen based on a purposive sample, and there will be between 3 and 6 observations completed. The portfolio lead will be asked to provide a list of all meetings taking place in the early stages of the program, which is likely to be within the first 6-9 months of the program. A sample of meetings most likely to demonstrate partnership working in practice [23] will be selected to be observed, such as planning meetings and advisory group meetings. The meetings will be chosen by the researcher to address any potential bias and ensure the appropriate independence of the research.

A framework for partnership working will be used to guide observations (Textbox 1 [24]). This model describes 4 key dimensions of partnership: process, actors (identity and position), decisions, and power relationships. Although the use of this framework provides some structure to the observations, a form of semistructured observation will be adopted to allow for some naturalistic observations [23] and include themes identified in the document analysis.

Nonparticipant observation will allow observation of the environment, language, nonverbal data, and interaction in partnership. General context will be noted for each observation, including location, time, duration, meeting roles, and purpose of the event or meeting.

There is a possibility that the presence of a researcher will increase the risk bias by changing the behavior of participants, and strategies will be used to reduce this risk. Strategies will include giving a clear explanation of the plan for observation and being aware of the position of the researcher to be as unobtrusive as possible [25]. Observations will be primarily descriptive and will provide the basis for the interpretation of data obtained by semistructured interviews in the final stage of data collection. Meetings will be held with the public partner to discuss themes developed at this stage of data collection and to agree on the format of semistructured interviews in phase 2.

**Framework for partnership working observation guide used in an exploratory case study, adapted from Carpentier.**

**Dimension of partnership working and observation guide**

- Process
  How is partnership working planned for and what preparations are in place to support partnership working?
  How many events or meetings involve people with lived experience?
  Who is involved in setting the agenda and context for meetings?
- Actors: identity and position
  Who attends meetings?
  What are people’s positions within the organization or program?
- Decisions
  How are decisions in the program made?
  How are people with lived experience involved in decision-making in the program?
- Power relationships
  Who contributes to the event or meeting?
  What is the response to people with lived experience’s contribution?
  What efforts are made to support contributions from people with lived experience?

#### Phase 2: Semistructured Interviews

The final stage of data gathering will be semistructured interviews with participants from the PD Improvement Programme, including people across disciplines and people with lived experience. Interviews will be used to gain an understanding of participants’ experiences and perceptions of partnership working with people with lived experience. A schedule for interviews will be prepared based on themes developed from the document review and observations. The interview proforma will be developed with people with lived experience working as a public partner in HIS to ensure questions are relevant and likely to receive meaningful responses [22]. All interviews will follow the schedule developed as an aide memoire; however, it is important to allow flexibility to adapt to each participant’s response to allow exploration of emerging and reported experiences [26]. Interviews will be held at a location agreed upon the researcher and participant and may be face-to-face, remote through Teams (Microsoft Corporation), or by telephone. All interviews will be recorded and transcribed.

The population within this case will include a purposive sample of staff and people with lived experience who are involved in and contribute to the work of the PD Improvement Programme. It is anticipated that this will be between 6 and 8 interviews. Participants will include clinical and improvement staff working directly on the PD Improvement Programme operating at different levels of the organization and people with lived experience working with the PD Improvement Programme. This should ensure diversity within the perspectives gained from the interviews.

### Recruitment Strategy and Informed Consent

Participants will be recruited through the PD Improvement Programme and will include a purposive sample of people involved in the program based on their role. All people involved in the program will be offered the opportunity to participate in this study and will be asked to sign a consent form and return it to the researcher at the start of each stage of the research.

There will be a process of ongoing consent for each phase of this research. In phase 1, each participant in the meetings observed will be asked to consent to the observation and recording during selected meetings and consent to being contacted for an interview at the second phase of research if appropriate. This will ensure each participant has a full understanding of the research, their role within it, the benefits and risks, and their right to withdraw from the research. Each participant’s consent will be documented in a written form they will be invited to sign before the meeting. Consent will be reviewed at the start of the meeting as a process of ongoing informed consent. If there are participants in the meeting who do not consent, their contribution to the meeting will be omitted during transcription. For meetings held online, participants who do not consent will be offered the chance to turn their camera off during the meeting and use the chat box for contributions if required. This may affect the understanding of the wider context of discussions, and therefore, efforts will be made to observe meetings with full consent.

In phase 2, people will be asked to consent to participate in semistructured interviews. Consent will be documented for each participant; they will be asked to sign a written consent form, and consent will be confirmed verbally at the start of each interview. Once consent is documented, the researcher will select a purposive sample of people who will participate in interviews based on their role in the program. All people who have given consent will be contacted to discuss the next steps, and interviews will be arranged with participants to ensure they take place at a suitable time and setting.

### Data Analysis

Data analysis will organize, find patterns, and elicit themes in the data to help deepen an understanding of partnership working within the national PD Improvement Programme. There are various mechanisms for quality assurance within this research, including the use of a reflexive field diary, discussions with supervisors, and member checking where participants can check transcriptions following observations and interviews. During analysis, there will also be regular meetings with a public partner working in HIS to review and discuss themes to check emerging findings and the researcher’s interpretation, as a form of participant validation to improve scientific rigor. A framework for data analysis is outlined in Table 1 [27].

**Figure 2:**
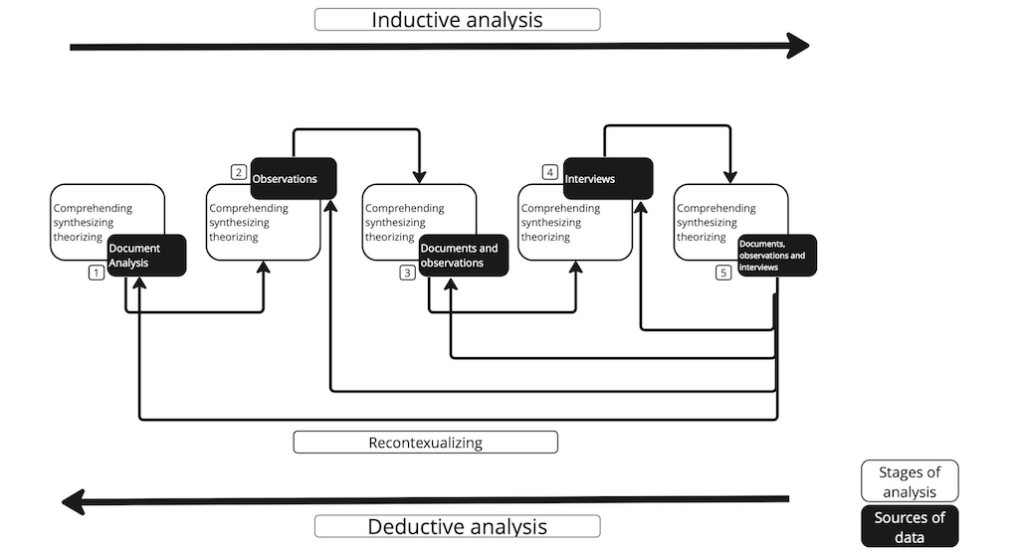
Data analysis plan for thematic analysis in an exploratory case study.

In order to develop convergent evidence, the structure outlined in Figure 2 will be applied to data analysis.

Effective organization of data will be important to this case study to enable the tracking of data sources, notes, documents, narratives, and other data [14]. NVivo (version 12; QSR International) will be used to support the management of data and to assist within and across case study analysis, appropriate to case study research [27]. Data collection and analysis will occur concurrently, as is practiced in qualitative studies [14].

**Table 1:** Thematic data analysis plan for an exploratory case study adapted from Houghton et al.

Stage of data analysis	Analysis strategy	Application for this research
Comprehending	Broad coding	This stage will analyze data to generate and develop codes. In this stage, enough data will be gathered to write a detailed, coherent, and rich description of partnership working.
Synthesizing	Pattern coding memoing	This stage will review codes identified at the broad coding stage and identify patterns within the data. Memos will provide summaries of key information for each theme, which will be used in further development of propositions of the data.
Theorizing	Distilling and ordering and testing executive summary statements	Relationships between categories of data will be examined, building a more integrated understanding of partnership working from all perspectives and data sources.
Recontextualizing	Developing propositions	Concepts identified will be synthesized to consider how the understanding of partnership working may be applied in different settings.

### Patient Involvement

The objective of this research is to deepen an understanding of how national improvement programs work in partnership with people with lived experience. This focus was developed through a review of current literature and organizational objectives [28] and has been highlighted by people with lived experience who have worked with HIS in other national MH improvement programs [29].

Patient involvement has been central to the development and design of this research, and a public partner has been involved in the design and will be involved in the analysis of this research. In phase 1, this included involvement in the review and analysis of themes as a form of participant validation to improve scientific rigor [30]. The public partner advised on the burden of intervention for people with lived experience in this study and has been involved in the design of phase 2, including the design of interviews, the development of the distress response policy, and advising on participant recruitment. The public partner will continue to be involved during the data analysis of phase 2, reviewing and discussing themes developed at this phase, and will be invited to advise on plans for dissemination of the study results to participants and linked communities.

### Ethical Considerations

Ethical approval has been granted from HIS’ research oversight group, the University of Stirling Research Ethics Committee, and the Integrated Research Application System through the Queen Square Research Ethics Committee (for phase 1; 318323) and the Black Country Research Ethics Committee (for phase 2; 309926). This study is part of a clinical doctorate program at the University of Stirling and is unfunded.

## Results

Data collection was completed in October 2023; analysis is expected to be completed and results published in January 2025.

## Discussion

This study will produce new knowledge on ways of working with people with lived experience and will have practical implications for all improvement focused interventions. Though the main focus of the study is on national improvement programs, it is anticipated that this study will contribute to the understanding of how all national public service organizations work in partnership with people with lived experience of MH care. The anticipated time for completion and write-up is 24 months. Information will be shared with key stakeholders on the progress of this research, including HIS and the University of Stirling, and opportunities for presentation of this research will be sought. These may include QI conferences and communities—including the Q Community (The Health Foundation), MH organization events, and NHS Scotland events. The findings will be completed with a thesis submitted to the University of Stirling and will be reported in an appropriate journal, such as *BMJ Open Quality* or the *Journal for Healthcare Quality*.

## Abbreviations

HIS: Healthcare Improvement Scotland
ihub: improvement hub
MH: mental health
PD: personality disorder
QI: quality improvement

## Data Availability

The data sets generated during and/or analyzed during this study are available from the corresponding author on reasonable request.
